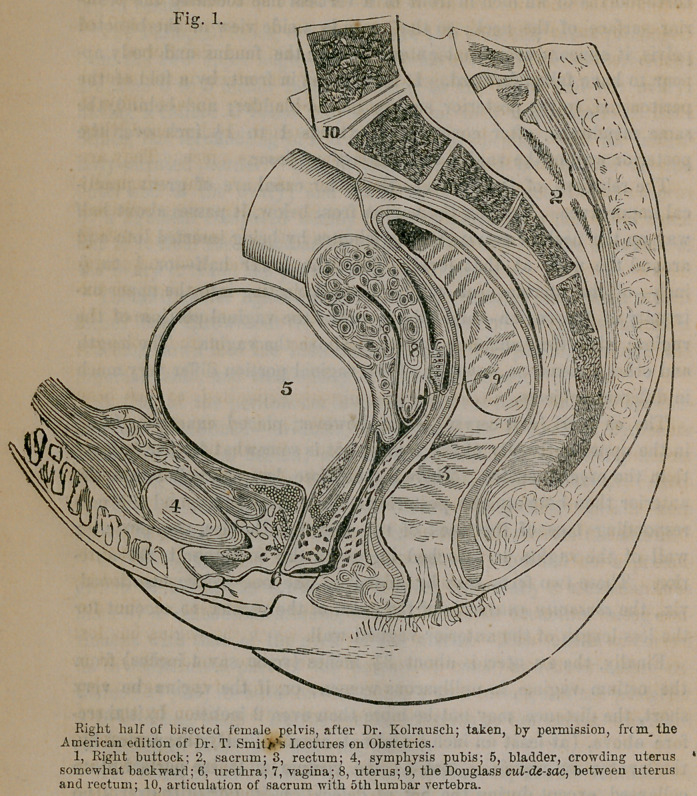# Lectures on Displacements of the Uterus

**Published:** 1860-03

**Authors:** E. R. Peaslee

**Affiliations:** Professor of Obstetrics and Diseases of Women and Children in the New York Medical College


### NEW YORK MONTHLY REVIEW

AND

### BUFFALO MEDICAL JOURNAL.

**VOL. 15.** MARCH, 1860. **NO. 10.**

### ORIGINAL COMMUNICATIONS.

*Lectures on Displacements of the Uterus.* By E. R. PEASLEE, M.D., LL.D., Professor of Obstetrics and Diseases of Women and Children in the New York Medical College.

(Delivered during the Session of 1859-60.)

GENTLEMEN—In conducting the Clinique for the Diseases of Women and Children during the present session, I shall first call your attention to displacements of the uterus. Some knowledge of the pathology and treatment of this class of diseases should be possessed by every practitioner; though, as a matter of fact, they are by a majority of our profession entirely overlooked. The clinique will constantly supply a large number of cases illustrative of the principles I shall adduce; and, avoiding all needless discussion, it will be my object, in as concise and familiar a manner as possible, to aid you in recognizing and appropriately treating the diseases under consideration. The other affections of the uterus and its appendages will receive attention in a subsequent part of the present course.

The displacements I have to consider are:

Prolapsus;
Retroflexion;
Anteflexion; and
Inversion.

But, before entering upon these respectively, certain preliminary topics present themselves; and to these alone the present lecture is devoted—viz.:

I. The structure of the uterus.

II. Its normal position and relations to other parts and organs.

III. The agencies which maintain it in position.

IV. I shall also consider, *in general*, the causes of displacements of the uterus. V. Their symptoms. VI. The methods of recognizing them; and VII. Their prognosis.

I. Of the *structure of the uterus* and its appendages, and of the vagina, I have given a detailed account in the first part of my course on Obstetrics. I shall here merely recapitulate such facts as are necessary to a correct idea of the class of cases I am about to bring before you.

The *uterus* is of a flattened, pyriform shape, and is divided into body and neck, (the upper part of the body being also termed the *fundus;*) the former being $1\frac{1}{2}$ to $1\frac{3}{4}$ inch, and the latter about $1\frac{1}{4}$ inch, long. The whole uterus, therefore, measures from $2\frac{1}{2}$ to 3 inches. It is $\frac{3}{4}$ to 1 inch thick, antero-posteriorly; and about 2 inches wide across the widest part of the body. The neck, or *cervix*, is $\frac{7}{8}$ inch to 1 inch thick. The whole organ weighs 7 to 10 drachms in the virgin state, and from 12 to 16 drachms in those who have borne children. After the child-bearing period has passed, however, the womb becomes gradually atrophied, and in the very aged woman becomes as small as in the girl before puberty.

In speaking of the *cavity* of the uterus, we must, as before, distinguish between the body and the neck. Indeed, for all practical purposes, and especially in treating of displacements, the body and the cervix must be regarded as distinct, though continuous, parts. The proper *cavity* of the uterus corresponds with the body merely—though in length only, and not in form. It is an isosceles triangle in outline, its apex merging into the upper extremity of the canal of the cervix below, while its other two angles extend above to the entrance of the oviducts, or Fallopian tubes. As the anterior and posterior walls of the uterus are very nearly in contact, the cavity, just bounded, can contain only a few drops (some say 15 to 20) of any fluid. It is lined by a layer called a mucous membrane; though it cannot be regarded as such on any physiological grounds, and should be regarded in its natural state merely as an undeveloped decidua, as I have explained to you. It is from $\frac{1}{2}$ to 1 line in thickness, whitish-red, and remarkable for the immense number of simple tubes, termed glands, which penetrate its entire thickness to the subjacent muscular layer.

The *canal of the cervix* is continuous with the cavity of the uterus just described, but different in all respects. It is about $1\frac{1}{4}$ inch long, is spindle-shaped, (the upper extremity being smaller than the lower,) and is flattened from before backward. Its upper end is termed the *orificium internum* of the uterus, and the lower is called the *os* uteri. The latter often, in the virgin state, but by no means always, terminating (as is seen through the speculum) in a curved outline, with a prominence of the neck both before and behind it—the term os tincæ, or tench's mouth, has been applied to it; and the prominences just mentioned are called respectively the anterior and the posterior lip of the os uteri. Very often, however, these two lips are not to be distinguished; the os being an opening in the centre of the cervix, while the latter is equally prominent on all sides, or is less prominent behind than before, as is more frequently the case. After parturition, also, the aperture of the os becomes transverse. You must, therefore, have no definite preconceived notion of the precise form of a patient's os uteri and its immediate surroundings, more than you would have of her nose, or any other feature of her countenance. Whether there be disease or not, you have therefore to decide, in very many cases, otherwise than by a reference to the precise conformation of this part. Anatomical treatises give you what may be termed the typical conformation; but to which only a small minority very accurately correspond.

The canal of the cervix is lined by a membrane entirely different from that lining the uterine cavity. It is folded into an immense number of laminæ, as Dr. Tyler Smith has demonstrated; the depressions between these offering a large secreting surface, which he terms an "open gland." Its clear, viscid secretion partakes of the properties of mucus, has an alkaline reaction, and to the eye much resembles the white of an egg. Just within the os uteri, and around it also, tactile papillæ are developed in considerable numbers, to which the sensibility in most women, of this surface, is due. The cervical canal and uterine cavity are together $2\frac{1}{4}$ to $2\frac{3}{4}$ inches long.

Finally, the uterus consists, histologically, of three layers of non-striated muscular fibre, arranged as I have before explained. These constitute the whole mass of the organ, (both body and neck,) except the lining membranes already described, and the peritoneum, which invests the body and a part of the neck of the organ externally. Vessels and nerves are abundantly distributed to the uterus; but of these I need not here give a particular description. I should, however, call attention to the fact that the walls of the uterus are thinnest—and especially the anterior wall—at the junction of the body with the cervix; and therefore it is at this point that flexions of the organ occur.

The Fallopian tubes, or *oviducts*, are two muscular tubes prolonged from the two upper angles of the uterine cavity, as before described; are from 3 to 5 inches long, extending to the right and left ovary; having a calibre or *lumen* only $\frac{1}{50}$th of an inch in diameter at their orifice, but terminating in a trumpet-shaped opening, with an irregularly-serrated border. One of these serratures is attached to the outer end of the ovary; and the latter is also attached to the uterus by a ligament composed mainly of muscular fibres, which thus reach out to it from the muscular layers of the uterus. Of the structure of the ovary it is not my present purpose to speak.

The two *round ligaments* are also two muscular arms, from 4 to 5 inches long, thrown out from the sides of the body of the uterus, each reaching down into the internal abdominal ring, passing through the inguinal canal, and terminating in the labia majora.

A fold of the peritoneum invests the uterus itself, and the appendages just mentioned—oviducts, ovaries, and the two sets of ligaments—both before and behind; and this fold, stretching completely across the pelvis, and inclosing these parts between its two layers, constitutes the *broad ligaments* of the uterus. They also contain some muscular fibres, extending between their two layers from the uterus.

On the other hand, the *vagina* may also be regarded as a prolongation of the uterus downward; it being essentially a muscular tube lined by a mucous membrane, and its muscular structure being continuous with that of the neck of the uterus. This tube is so curved as to constitute a part of the parturient canal, as I have before explained to you; its anterior wall being 4 to 5 inches, and the posterior 5 to 6 inches, long. But its precise relations to the cervix uteri will be explained under the following head.*

II. What is the *natural position of the uterus*, and its relations to other parts and organs?

Placed between the two layers of peritoneum constituting its lateral ligaments, the uterus is situated in the adult female very nearly in the axis of the superior strait of the pelvis; its fundus rising to a point just below the level of its superior plane. Its position is, however, not precisely vertical; but it is slightly curved anteriorly—this

* For the full particulars respecting the histology of the uterus and its appendages, I refer to my work on "Human Histology," pp. 559–566.

curve corresponding with the axis of the parturient canal, as I have previously explained to you. Thus, the fundus of the uterus is about three-fourths of an inch in front of a vertical line touching the posterior surface of the neck; so that, seen in a side view of the bisected pelvis, it appears somewhat anteverted; or the fundus and body appear to have fallen forward. It is attached in front, by a fold of the peritoneum, to the posterior surface of the bladder; and behind, the same membrane, after covering it, extends 1 to 1½ inch over the posterior wall of the vagina to Douglass' *cul-de-sac.*

The relations of the uterus to the latter canal are of great practical importance. Tracing the vagina from below, it passes about half way up the neck or cervix, and terminates by being inserted into and around the neck at that level. Thus, the lower half—or ½ to ¾ inch—of the uterine neck will be found projecting into the upper extremity of the vagina—and this is called the vaginal portion of the vagina; while the remaining portion is above the vagina. The length and other dimensions, however, of the vaginal portion differ very much in different subjects.

The os uteri (and cervix) is not, however, placed exactly *centrally* in the upper extremity of the vagina, but is somewhat farther in front than the precise centre. There is, therefore, less space between the anterior than between the posterior wall of the vagina, and the corresponding face of the neck of the uterus. Besides, the posterior wall of the vagina is attached higher on the neck than is the anterior. These two facts may be added to the one before mentioned, viz., the *concavity anteriorly* of the canal of the vagina, to account for the less length of the anterior vaginal wall.

Finally, the os uteri is about 3½ inches (some say 4 inches) from the ostium vaginæ, in nulliparous women; or, if the vagina be very short, the distance may not be more than even 2 inches. It is therefore above, (at least an inch,) and not at all directly supported by, the levator ani muscle. And, as the rectum is in its normal state collapsed, except during the act of defecation, it normally exerts no indirect pressure on the cervix. The accompanying diagram illustrates the relations of the parts just described.

Such are the normal position and relations of the uterus, in the nulliparous woman. But I should add, that in many who have borne children, this organ has a considerable latitude in these respects; the position varying in various ways, within certain limits from the account I have given; though these variations may produce no symptoms, and require no treatment. Not seldom, the same uterus will be found in a particular position at one examination, and in another a day or two subsequently.

Fig. 1.

Right half of bisected female pelvis, after Dr. Kolrausch; taken, by permission, from the American edition of Dr. T. Smith's Lectures on Obstetrics.

1, Right buttock; 2, sacrum; 3, rectum; 4, symphysis pubis; 5, bladder, crowding uterus somewhat backward; 6, urethra; 7, vagina; 8, uterus; 9, the Douglass *cul-de-sac*, between uterus and rectum; 10, articulation of sacrum with 5th lumbar vertebra.

III. How is the uterus maintained in the position which has just been specified?

1. The *direct* supports of the uterus are the broad, the round, and the utero-rectal ligaments.

The *broad* ligaments, already described, in their normal condition, keep the body of the uterus from falling directly forward, or directly backward or downward. Indeed, if they do not yield at all, they may serve to prevent displacement of the body of the uterus in any direction. But, as they stretch completely across the pelvis, and inclose the uterine body in their central position only, it is evident that if they become relaxed or elongated by any cause, they so far cease to fulfill this function, and allow more or less displacement of the inclosed organ, in any and in all directions; and their tonicity will of course vary at different times, since they contain muscular fibres.

The *round* ligaments maintain the body of the uterus in its natural slightly anteverted position; or, in other words, they do not allow it to fall backward from that position. If stretched, or shortened, they of course fail proportionately to fulfill their precise office. They are also essentially muscular—containing some striated, but principally non-striated, fibres; and therefore vary in their contractile conditions.

The *utero-rectal* ligaments are mainly folds of peritoneum, extending backward from the sides of the womb to the rectum; and these ligaments prevent the lower part of the body and the upper part of the cervix from coming forward beyond a certain distance (about 1½ inch) in front of the sacrum. They form the sides of the *cul-de-sac*, Fig. 1, 9.

The *cervix* of the uterus is also directly supported, as has been shown, by the posterior wall of the bladder, and by the vagina also, provided the latter maintains its position. It can, however, accomplish this, and support the cervix, only in virtue of its contractile power: *i. e.*, if, being a muscular tube, it contracts so firmly as not to allow the uterus to descend into it, (provided its other supports, before mentioned, allow it to descend,) it directly supports it; but if its contractile force is essentially diminished, it renders no such aid as has been ascribed to this canal, in preventing the descent of the uterus. On the other hand, in many cases in which the vagina has lost its tone, the other direct supports of the uterus are found sufficient to sustain it and its appendages, and the upper portion of the vagina also.

2. The uterus is also *indirectly* supported in its position by the rectum, the levator ani muscle, and the perineum. By this I mean to say, that if the direct supports of the uterus fail, these parts will arrest its tendency downward towards the os externum; or that, if these parts, on the other hand, lose their natural position or their force, the direct supports before mentioned may not alone be found sufficient to maintain the womb in position, and displacement, therefore, ensues. Thus, prolapsus uteri often occurs in consequence of prolapsus of the rectum, rupture of the perineum, or loss of tone of the levator ani. The important relations of this muscle in the female will be discussed in connection with prolapsus of the uterus.

IV. The *causes* of displacements of the uterus are therefore, in general, as follows:

1. Agencies which weaken the direct supports of the uterus. And since the latter are principally muscular in structure and in action, I may mention, under this head, general debility, (as in anæmia,) relaxation after delivery, and great efforts, even in the unmarried, especially during the monthly period.

2. Any agency enfeebling the indirect supports above mentioned. And here should be mentioned especially the effects of child-bearing in relaxing the levator ani, and the other parts included in this class.

3. Any cause increasing the *weight* of the uterus itself, and its appendages: *e. g.*, congestion, inflammation, hypertrophy, induration, scirrhus, fibrous tumors, or polypus of the uterus; moles, hydatids, and early pregnancy.

4. Pressure upon the uterus by displaced or enlarged contiguous organs, (bladder or rectum;) by tumors of the pelvis or abdomen, ascites, and ovarian disease.

V. The *symptoms* of the various displacements of the uterus are by no means so distinctive as is often supposed. Some, and often many, of the following symptoms are common to them all, and to several other uterine affections: A feeling of fullness in the pelvis; a bearing-down, a dragging, or an aching sensation in the umbilicus, hypogastrium, pelvis, loins, sacrum, nates, groins, or thighs; frequent or difficult micturition; constipation and tenesmus; a tenderness of the cervix uteri on pressure, especially of its posterior portion; and some derangement of menstruation, and a leucorrhœal discharge. Subsequently, also, the stomach becomes deranged, and the bowels inactive; the appetite diminishes, and the spirits are depressed. Not seldom, these latter symptoms occur without having been preceded by the former to any marked extent, and their true cause would hardly occur to one not familiar with this class of ailments. In such cases, the poor patient too often receives but little sympathy from her friends, and perhaps from her husband, even; being regarded as merely nervous, hysterical, or hypochondriacal—terms which, in common parlance, are used to cover cases which are supposed to have no cause but in the imagination, and which are assumed to be attended by none but imaginary suffering. But if any person more than any other deserves, and also actually needs, sympathy, it is a woman who thus suffers from a cause unsuspected by herself, or knowing which, she must still conceal it from those around her.

We can therefore very seldom decide with any degree of assurance, in any given case, from the rational signs merely, that any particular displacement exists rather than another; or, indeed, that we have a case of displacement, rather than of inflammation or ulceration, or some other affection still, of the uterus. We can obtain a correct diagnosis with certainty, only by means of an internal examination; and to this topic we next devote our attention. To the *rational* signs I shall therefore give but little prominence, when I come to speak of these displacements in detail.

VI. Uterine diseases are, at the present day, diagnosticated, as you are aware, mainly by the touch, the speculum, and the uterine sound.

The *speculum* is invaluable, in its proper use; but it has no place in the diagnosis of mere displacements of the uterus. I have explained its value in the diagnosis and treatment of certain other affections; and which I could not conscientiously treat, at the present day, without its aid. But it should be a rule never to use it unless absolutely necessary; and therefore I have no more to say of this instrument in connection with my present subject.

But the *uterine sound*, invented a few years since by Prof. Simpson, of Edinburgh, here finds its appropriate sphere. Indeed, there are many cases of deviation from the normal position of the uterus which cannot possibly be recognized without it. In all doubtful cases, therefore, the sound should be used; but with this proviso—*it is never to be used in any case, unless we previously assure ourselves, from positive reasons and testimony, that the patient is not pregnant.*

Some very frightful stories have been told of the danger to the female from passing a sound into the uterus; but you have seen the operation so often performed at the clinique with entirely negative results, so far as any sense of injury is concerned, that you will hardly credit them. It is not to be denied, however, that there is sometimes a degree of hyperæsthesia of the uterine lining membrane which renders the introduction of the sound exceedingly painful; and in a single instance, you will recollect that a patient nearly fainted from the pain and shock, but very soon rallied, and walked home without any difficulty. The simplest operation sometimes produces unusual and unexpected results. The passage of a bougie into the bladder of a male has been known to produce even a fatal shock. Still, no one hesitates to perform that operation in circumstances requiring it. I should say that, generally, the uterine sound produces far less suffering, and subsequent irritation, than the vesical catheter; and I have never in a single instance witnessed any serious symptoms after its use which I could attribute to it alone. Of course much harm can be done with it, if guided by a reckless head and hand; and the utmost delicacy in its application is essential.

Of the *touch* I need only say, it is also indispensable to the diagnosis of these displacements. It is usually performed by the index finger alone, and in a manner with which you have been made familiar. There is an advantage in habitually applying it with the same index finger, whether the right or the left, as the sense becomes thus more highly developed and educated for such purposes. I have found much advantage from using the left forefinger for delicacy of touch, and the right in all cases requiring the exertion of strength.

VII. Further, I have a word to say on the *prognosis* of uterine displacements. Those who have had much experience in their treatment will not be too sanguine of a complete cure in most cases, unless the patient can be kept in the most favorable circumstances, and for a sufficient length of time; and this is, in a majority of cases in private practice, quite impossible. We can, however, almost always expect to secure much alleviation of the symptoms, and very often expect to remove them entirely. But we can in no case guarantee exemption from a relapse, if a complete cure is effected; since the same causes may reproduce the disease, and especially in the married.

Do not understand me to underrate the benefits of appropriate treatment in the displacements under consideration; for I could not mention another department of medical practice in which so much suffering is removed as by their appropriate management. I only wish to caution you against that enthusiasm which often, in a young or an ignorant practitioner, would promise a complete cure in circumstances or in cases which admit only of more or less relief. The more we have to do with this class of cases, the more sanguine shall we be of affording vast relief, and the less of a complete and permanent recovery. You will therefore not, I trust, adopt the custom of some who promise a cure for a certain sum of money paid in advance.

Of the treatment calculated to insure the best results, I will speak in connection with each particular displacement.

## Figures and Tables

**Fig. 1. f1:**